# Supercooled storage of red blood cells slows down the metabolic storage lesion

**DOI:** 10.21203/rs.3.rs-5256734/v1

**Published:** 2025-02-27

**Authors:** Travis Nemkov, Ziya Isiksacan, Nishaka William, Rahime Senturk, Luke E. Boudreau, Martin L. Yarmush, Jason P. Acker, Angelo D’Alessandro, O. Berk Usta

**Affiliations:** 1 Department of Biochemistry and Molecular Genetics, University of Colorado Anschutz Medical Campus, Aurora, CO, USA 80045.; 2 Center for Engineering in Medicine and Surgery, Department of Surgery, Massachusetts General Hospital, Harvard Medical School, Boston, MA 02114.; 3 Shriners Children’s, Boston, MA 02114.; 4 Laboratory Medicine and Pathology, University of Alberta, Edmonton, AB T6G 2R8, Canada.; 5 Department of Chemical Engineering and Chemistry, Eindhoven University of Technology, Eindhoven, The Netherlands, 5612 AZ.; 6 Department of Biomedical Engineering, Rutgers University, Piscataway, NJ, USA, 08854.; 7 Innovation and Portfolio Management, Canadian Blood Services, Edmonton, AB T6G 2R8, Canada.

## Abstract

Red blood cell (RBC) transfusion, a life-saving intervention, is limited by reduced RBC potency over time. Cold storage at +4 °C for up to 42 days can reduce transfusion efficacy due to alterations termed the “storage lesion.” Strategies to mitigate the storage lesion include alkaline additive solutions and supercooled storage to extend storage by reducing metabolic stresses. However, RBC metabolism during supercooled storage in standard or alkaline additives remains unstudied. This study, thus, investigated the impact of storage additives (alkaline E-Sol5 and standard SAGM) and temperatures (+4 °C, −4 °C, −8 °C) on RBC metabolism during 21- and 42-days storage using high-throughput metabolomics. RBCs stored with E-Sol5 showed increased glycolysis and higher ratios of reduced to oxidized glutathione compared to SAGM. Supercooled storage at −4 °C showed markedly lower hemolysis than −8°C, preserved adenylate pools, decreased glucose consumption, and reduced lactate accumulation and pentose phosphate pathway activation. The combination of supercooled storage and E-Sol5 helped to preserve ATP and 2,3-DPG reservoirs, while preventing catabolism and free fatty acid accumulation. While supercooled storage with E-Sol5 offers a promising alternative to standard storage, preserving RBC metabolic and functional quality, further research is necessary to validate and improve on these foundational findings.

## Introduction

Red blood cell (RBC) transfusion, facilitated by the donation of 120 million units globally, is a common therapeutic procedure^1–3^. Amidst the ongoing discussions on implementing restrictive transfusion regimens, declines in blood donations – especially due to the COVID-19 pandemic – combined with increased demands due to recent regional wars have further underscored the constraints associated with blood shortage. Such crises fueled once again the need and search for novel storage strategies to facilitate improved logistics, including approaches to extend the shelf-life for RBCs. Today, packed RBC units are stored under refrigerated conditions (+4 °C, a.k.a. cold storage) for up to 42 days in the United States. RBC cold storage is associated with an estimated 17% loss of transfusion potency at outdate upon transfusion^4^. Such estimates are based upon quality assessment as per Food and Drug Administration (FDA) gold standards of in-bag hemolysis (<1%) and extravascular hemolysis, the latter determined via quantitation of post-transfusion circulatory capacity of stored, radiolabeled RBCs at 24h after transfusion (>75%)^5,6^. Recently, both the FDA and the United States National Heart, Lung and Blood Institutes (NHLBI) have underscored the limitations of these measurements^7^. Specifically they have highlighted that current measures are insufficient to capture the complexity of the extensive biochemical, morphological, and functional alterations RBCs undergo as a function of storage duration, collectively referred to as the storage lesion^8,9^.

Extensive literature has documented the principles behind the onset, progression, and ultimate severity of the storage lesion during cold storage^9^. For example, cold storage negatively impacts the activity of cation transporters, promoting extracellular potassium accumulation and intracellular influx of calcium and sodium^10^. While slower than at 37°C, metabolism at 4°C favors the intracellular acidification, in turn inhibiting pH-sensitive enzymes at rate-limiting steps of glycolysis, the Rapoport-Luebering shunt, and the pentose phosphate pathway (PPP)^11,12^. Consequently, bisphosphoglycerate phosphatase activity is favored over the kinase activity and leads to the depletion of 2,3-diphosphoglycerate (2,3-DPG), which in turn negatively impacts oxygen binding and off-loading kinetics^13,14^. Uncontrolled accumulation of reactive species targets redox-sensitive rate-limiting steps including glyceraldehyde 3-phosphate dehydrogenase^15^, which favors PPP utilization to generate NADPH reducing equivalents^9,16,17^. However, this response is insufficient to preserve reduced glutathione pools, which decline steadily during storage^18^, in the face of insufficient de novo synthesis^19^ and exacerbated catabolism into 5-oxoproline^20^ and glutathionylated oxylipins^21^. Consequently, irreversibly oxidized proteins and membrane lipids are progressively vesiculated from the RBCs. These events initiate morphological alterations that compromise RBC viscoelasticity and induce osmotic fragility^22–25^, ultimately promoting extravascular splenic sequestration and erythrophagocytosis by residential macrophages^26^. The cumulative effect of these lesions is the reduced stored cell quality and, potentially, transfusion efficacy and safety.

While clinical retrospective studies have emphasized the increased risks of transfusing RBC units older than two weeks compared to fresh units^27–29^, reassuring evidence from multiple randomized clinical trials (reviewed in^30^) suggests that current standards of practice are non-inferior to the selective transfusion of the freshest available blood^31,32^. Still, it has become increasingly evident that blood, like its human donors, does not always age the same, and the chronological age of a blood unit (days after donation) does not equate to its molecular age (as characterized, for example, by omics phenotyping^33^). The storage lesion is exacerbated in some categories of donors as a function of biology (sex, age)^34^, genetic traits^35–38^, and lifestyle-associated exposures^39^ (comprehensively reviewed in^22,34,40,41^). In this view, the NHLBI recommended that the development of improved RBC storage practices be guided by omics phenotyping^8,42,43^, as we performed in the present study.

Historically, the efforts to improve RBC storage centered on improving the additive solutions through compositional variation^44^, at +4 °C^44^. The early class of additive solutions like SAGM (saline, adenine, glucose, and mannitol) are acidic. While RBCs rapidly buffer these solutions upon exposure, these additives promote the intracellular acidification. In contrast, novel additive including phosphate-adenine-glucose-guanosine-gluconate-mannitol (PAG3M), Erythro-Sol 5 (E-Sol5), and SOLX (AS-7) have pursued different strategies to allow RBC storage under alkaline conditions^45,46^. These solutions are chloride-free (or low chloride) to enable chloride shift (influx of the weak acid bicarbonate in exchange for the efflux of the strong chloride). The influx of OH^−^ via Donnan equilibrium further skews the intracellular pH towards alkalinity, predominantly in the first week of storage^46–48^. Despite the benefits of storing RBCs in these novel formulations, conventional solutions are still in use (SAGM in Europe and Canada and CPDA-1 in the United States). This is partly due to the poor logistics associated with sterilization of high glucose containing alkaline additives, a process that requires separation of the high sugar fraction from the alkaline component to prevent caramelization of the former at high temperature. Storage by freezing of glycerolized RBCs has been pioneered in the ‘70s by Valeri^49^, a protocol that is now established to store rare blood types, but is not routinely practiced due to complex processing steps and equipment^50^. The feasibility of the implementation of frozen blood storage for the full inventory has been challenged upon the tragic events at the Twin towers in 2001 in New York, when massive amounts of blood were urgently needed, yet the frozen units were not readily available owing to the lengthy logistics associated with thawing and deglycerolization^51^. Thus, despite the well-established benefits of frozen storage for the metabolic and morphological storage lesion^52–54^, novel storage methods are needed to overcome the current logistical limitations of this practice at scale.

We had recently proposed supercooled storage of RBCs as a promising alternative to the standard cold or frozen storage practices^55^. We demonstrated the feasibility of this approach with diluted RBCs (<0.1% hematocrit) at temperatures as low as −13 °C without any phase change^55^, where ice nucleation was inhibited by sealing the liquid-air interface with hydrocarbon-based liquids^56,57^. Later, we increased the hematocrit to ~50% and tested additive solutions for their ability to maintain low hemolysis during supercooled storage of RBCs between −4 to −8 °C^4,58^. RBCs in E-Sol5 underwent significantly lower hemolysis than in all other solutions (SAGM, CPDA-1, PAG3M, AS-7) at any time both at the cold (+4 °C) and supercooled storage temperatures^58^. Conversely, using SAGM resulted in significantly higher hemolysis at all temperatures, especially in the supercooled range.

These recent studies showed the premise of supercooled storage for long-term storage of RBCs^4,58^. Yet, there remains a significant gap in our understanding of how and why different storage temperatures (cold and supercooled) and times, and additive solutions affect the storage lesion and RBC metabolism. Such understanding is critical to improving this promising supercooled RBC storage beyond using additive solutions optimized for cold storage. Thus, here, we investigated the changes in metabolic phenotypes of RBCs during storage for up to 42 days and tested the impact of storage additives (alkaline – E-Sol5 or standard – SAGM) and temperatures (+4 °C, −4 °C, −8 °C).

## Materials and Methods

### Inclusion and ethics statement:

All sample preparation and experiments other than the metabolomics were performed at the Massachusetts General Hospital (MGH). The study was approved by the Institutional Review Board of MGH (protocol number: 2019P003498) and performed in accordance with all the relevant ethical regulations and guidelines.

### Experimental materials:

All additive solution compounds were purchased from Sigma-Aldrich (MA, USA). Round-bottom polypropylene centrifuge tubes and polystyrene serological pipets were purchased from Genesee Scientific (CA, USA). Clear flat-bottom polystyrene 96-well plates and Falcon 5 ml round-bottom polystyrene tubes were purchased from Corning (MA, USA). Covidien polypropylene specimen containers (120 mL) were purchased from Fisher Scientific (MA, USA). V-bottom polypropylene 96-well plates and aluminum well plate sealers were purchased from Greiner Bio-One (NC, US).

### Solution preparation:

Fresh additive solutions were prepared for each experiment as reported previously^58^. The pH of additive solutions was adjusted as pH=8.4 for E-Sol5 and pH=5.5 for SAGM. Solutions were then filtered through a vacuum filter system with a 0.22 μm polyethersulfone membrane (25–227, Genesee Scientific, CA, USA).

### Blood processing:

Packed human RBCs in CPDA-1 (~180 mL) in polyvinyl chloride bags from 4 donors were purchased from Research Blood Components, LLC (MA, USA). The company obtained informed consent from the 2 female and 2 male healthy donors. RBC units were received the day they were collected and stored at +4 °C until clearance for viral infection, upon which they were processed for storage within 24 hours. No pooling was performed. Each RBC unit was transferred to a sterile container, split at a volume of 20 mL into 50 mL conical tubes, and then suspended in 15 mL of an additive solution (E-Sol5 or SAGM). The samples were centrifuged at 1,500xg and washed with the corresponding additive solutions at ~50% hematocrit levels 3 times. Then, the samples were kept at +4 °C overnight and centrifuged again at 1,500xg, and washed with the corresponding additive solutions at a final hematocrit of ~50%. The samples were aliquoted as 1 mL units in 5 mL polystyrene tubes and capped with 0.5 mL mineral oil^58^. These units were stored in temperature-controlled freezers (Engel MHD-13, Engel, FL, USA). For each donor, the samples were split into experimental groups according to the additive solution (E-Sol5 or SAGM) used, the storage temperature (+4 °C, −4 °C and −8 °C), and the storage time (day 0, day 21 and day 42). For each experimental group, 3 samples were prepared resulting in 216 total samples (4 (donors) × 2 (solutions) × 3 (temperatures) × 3 (timepoints) × 3 (technical replicates)). At the designated timepoints, stored samples were transferred to 1.5 mL Eppendorf tubes and centrifuged at 2000xg for 10 min to separate the pellet from the supernatant. Pellets and supernatants were transferred into V-bottom 96-well plates at sample volumes of 150 μL on dry ice. The plates were then sealed and stored frozen at −80 °C until shipment for metabolomics analysis.

### RBC quality assays:

Hematocrit levels were measured using an automated hematology analyzer (XP-300, Sysmex, IL, USA). Hemolysis was evaluated through absorbance at 540 nm with a Drabkin’s-based method to determine the ratio of supernatant to total hemoglobin by a plate reader (SpectraMax iD3, Molecular Devices, CA, USA) with a correction for hematocrit. Drabkin’s reagent was prepared in-house as previously reported^59^.

### High-throughput metabolomics:

Metabolomics extraction and analyses in 96 well-plate format were performed as described previously^60^. The frozen samples in V-bottom 96-well plates at MGH were transferred in dry ice to the University of Colorado Anschutz Medical Campus. Plates were thawed on ice, and then a 10 μL aliquot was transferred with a multi-channel pipettor to 96-well extraction plates. 90 μL of ice cold 5:3:2 MeOH:MeCN:water (*v/v/v*) was added to each well, with an electronically-assisted cycle of sample mixing, repeated 3 times. Extracts were transferred to 0.2 μm filter plates (Biotage, NH, USA), and the insoluble material was removed under positive pressure using nitrogen applied via a 96-well plate manifold. Filtered extracts were transferred to an ultra-high-pressure liquid chromatography (UHPLC-MS — Vanquish, Thermo Fisher Scientific, MA, USA) equipped with a plate charger. A blank and a quality control sample (the same across all plates) were injected 2 or 5 times each per plate, respectively to monitor instrument performance. Metabolites were resolved on a Phenomenex Kinetex C18 column (2.1 × 30 mm, 1.7 um) at 45 °C using a 1-minute ballistic gradient method in positive and negative ion modes (separate runs) over the scan range 65–975 m/z as previously described. The UHPLC was coupled online to a Q Exactive mass spectrometer (Thermo Fisher Scientific, MA, USA). The Q Exactive MS was operated in negative ion mode, scanning in Full MS mode (2 μscans) from 90 to 900 m/z at 70,000 resolution, with 4 kV spray voltage, 45 sheath gas, and 15 auxiliary gas.

### Metabolomics Data Analysis:

Following data acquisition, raw files were converted to mzXML using RawConverter^61^. Metabolite assignments and peak integration were performed using El-Maven (Elucidata, San Francisco, CA, USA) in conjunction with an in-house standard library^62^. Multivariate analyses of processed metabolomics data were performed with MetaboAnalyst 6.0^63^. For normalization to day 0 calculations, missing values were imputed with 20% of the median value for a given metabolite across the entire dataset. Individual metabolites were graphed with GraphPad Prism 10.2.2 (GraphPad Software, Inc., MA, USA).

## Results

### Additive solutions and storage temperature have significant impacts on stored RBC metabolism

**Figure 1.**
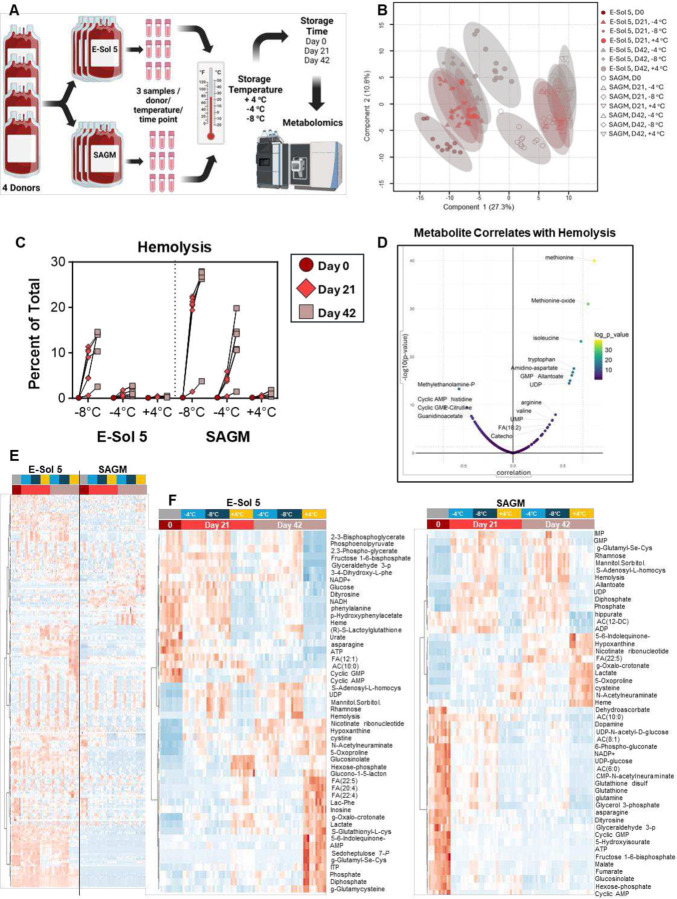
Metabolomics of Supercooled Storage. (A) Red blood cells from 4 donors were suspended (in technical triplicate) in either SAGM or E-Sol5 and stored at +4°C, −4°C, or −8°C. Samples were taken prior to temperature adjustment at day 0, or after 21 and 42 days in respective storage conditions. RBC and supernatant fractions were analyzed by LC-MS metabolomics. (B) Partial-Least Squares Discriminant Analysis (PLS-DA) of the RBC fraction. (C) Hemolysis measures (in % of total) at the respective time points (day 0, ○; day 21, ◊; day 42, ☐) at each of the temperatures in E-Sol5 or SAGM. (D) Pearson correlations between all RBC metabolites and hemolysis metrics with respective p-value provided by color code. (E) Hierarchical clustering analysis (HCA) of all RBC metabolites measured in all storage conditions. (F) HCA depicting top 50 ANOVA significant metabolites in E-Sol5 (left) and SAGM (right) storage.

The experimental design is illustrated in Fig 1A. Briefly, RBC units from 4 donors (2 males and 2 females) were split and stored in either E-Sol5 or SAGM additive solutions at +4, −4, or −8 °C. Samples were collected at days 0, 21, and 42 for metabolomics analyses. Unsupervised analyses of metabolomics and functional data (*i.e.*, hemolysis) are shown in the form of a partial least squares discriminant analysis in Fig 1B, which shows that storage temperature and additives (across PC1 – explaining 27.3% of the total variance) and time (PC2 – 10.8%) had a significant impact on RBC parameters. The highest hemolysis was observed for units stored in SAGM, both at −8 and - 4 °C, followed by E-Sol5 at −8 °C (Fig 1C), suggesting a protective effect of the alkaline additive for supercooled RBCs. These results also reveal a negligible difference in hemolysis between supercooled RBCs stored in E-Sol5 and RBCs stored in SAGM at +4 °C. Notably, the levels of several amino acids positively correlated with hemolysis across all conditions, with methionine and its oxidized metabolite (methionine oxide) showing the strongest positive correlations (Pearson R > 0.6, −log10(p) > 30 – Fig 1D), followed by isoleucine, tryptophan, arginine, and valine. Negative correlations were instead noted between hemolysis and basic amino acids histidine and citrulline, and cyclic AMP and cyclic GMP. Altogether, this analysis suggests a previously unreported, temperature/additive-dependent association between stored RBC hemolysis and amino acid levels or adenylate/guanylate cyclase activities.

The summary heat map of the significant metabolites by ANOVA (by storage temperature, adjusted by time and storage additive – Fig 1E) shows a dramatic impact of the two additive solutions and a significant difference in metabolism during storage due to their use. This warranted a detailed breakdown of the metabolomics results for the two solutions tested, E-Sol5 and SAGM as shown in Fig 1F.

### E-Sol5 boosts RBC metabolism, while supercooled preservation slows down catabolism

**Figure 2.**
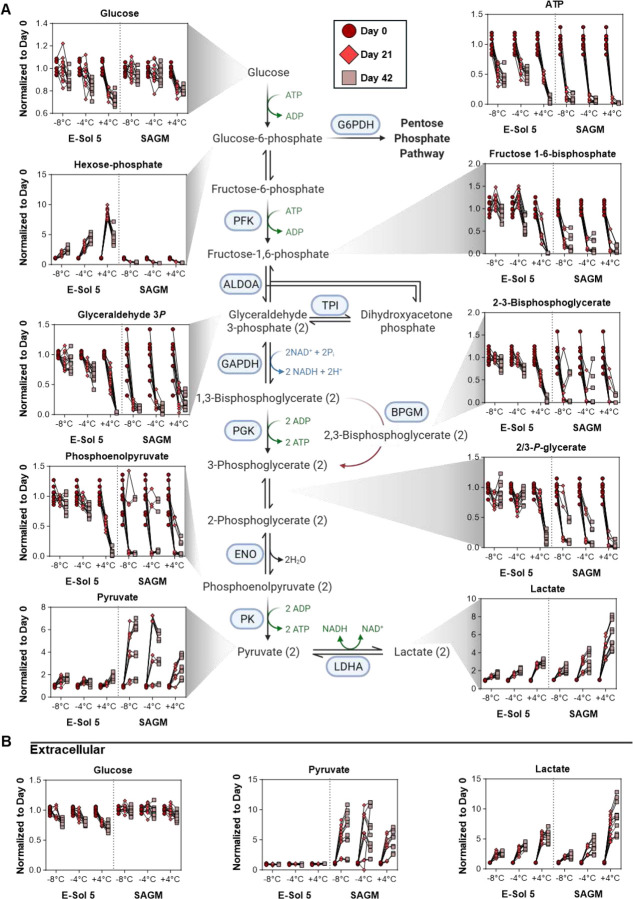
Glycolysis. Relative abundance measurements (normalized to respective day 0 value for each donor) for glycolytic metabolites for both (A) intracellular and (B) extracellular compartments at day 0 (○), day 21 (◊), and day 42 (☐). Samples from individual donors are connected by lines.

RBCs stored in E-Sol5, independently from storage temperature, were characterized by significantly higher intracellular glucose (**Supplemental Figure 1**). This likely stemmed from the higher substrate availability in E-Sol5, which also resulted in increased steady-state levels of all glycolytic intermediates throughout the storage. Storage-dependent depletion of glycolytic intermediates downstream of fructose 1,6-bisphosphate was observed in all temperature groups (Fig 2A), albeit more slowly in supercooled RBCs. Of note, RBCs stored in E-Sol5 preserved 2,3-DPG levels throughout storage better than those in SAGM at 4 °C. More importantly, the 2,3-DPG breakdown was reduced in supercooled RBCs both at −4 and −8 °C (Fig 2A). Lactate generation was ultimately the highest in E-Sol5 both in cells and supernatants (**Supplemental Figure 1**). However, the relative rate of lactate accumulation was higher in SAGM (Fig 2A–B). Slower glycolytic fluxes at −4 and −8 °C in both solutions, inferred from steady-state levels of glycolytic intermediates, confirm that supercooled temperatures slow down metabolic activity (Fig 2A). Continued, albeit slower, changes in metabolites in the supernatants indicate that metabolic activity does not cease at supercooled temperatures (Fig 2B). Importantly, we observed significantly better preservation of ATP pools at supercooled temperatures (Fig 2A). In summary, while E-Sol5 boosts RBC glycolysis compared to SAGM, supercooled storage significantly slows RBC metabolism, leading to low lactate production and minimal depletion of 2,3-DPG and ATP pools.

### Supercooled storage alleviates transient oxidant stress-driven activation of the oxidative phase of the pentose phosphate pathway

**Figure 3.**
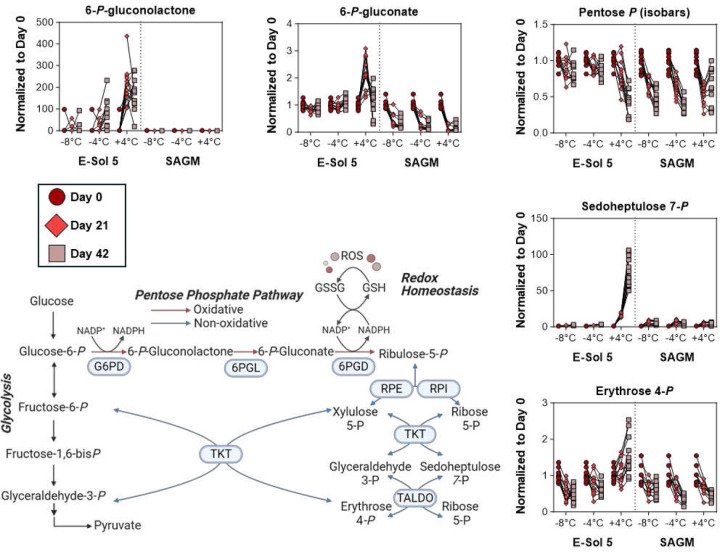
Pentose Phosphate Pathway. Relative intracellular abundance measurements (normalized to respective day 0 value for each donor) for pathway intermediates at day 0 (○), day 21 (◊), and day 42 (☐). Samples from individual donors are connected by lines.

It has been previously reported that a blockade in glyceraldehyde 3-phosphate (G3P) metabolism by Glyceraldehyde 3-phosphate dehydrogenase (GAPDH) oxidation promotes the transient activation of PPP^15^. This results from G3P accumulation upstream and shunting of hexose monophosphates to the oxidative phase of the PPP to generate nicotinamide adenine dinucleotide phosphate (NADPH), which in turn fuels antioxidant systems. Consistent with the literature, depression of late glycolysis downstream of phosphofructokinase is observed at +4 °C even in the more metabolically active RBCs stored in E-Sol5 (Fig 2A). Accordingly, elevations in 6-phosphogluconolactone, 6-phosphogluconate, and sedoheptulose 7-phosphate are observed in this same group as a function of storage time and additives (highest in E-Sol5 RBCs at +4 °C – Fig 3). Conversely, supercooled RBCs show no notable change in late PPP end products (pentose phosphate isobaric isomers) and sedoheptulose 7-phosphate (Fig 3) when stored in E-Sol5, but not in SAGM. Altogether these results suggest that elevated oxidant stress promotes redirection of fluxes to the PPP, especially at +4 °C, a phenomenon reversed by supercooled storage in E-Sol5 but not SAGM.

### E-Sol5 and supercooled storage boost the ratios of reduced to oxidized glutathione, while only supercooled storage prevents cystine efflux

**Figure 4.**
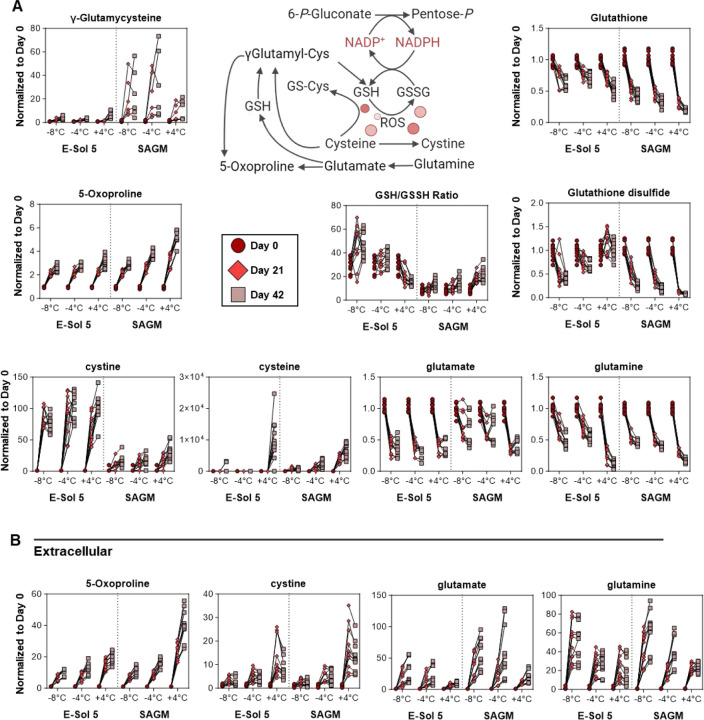
Glutathione Homeostasis. Relative abundance measurements (normalized to respective day 0 value for each donor) for glutathione and associated metabolites for both (A) intracellular and (B) extracellular compartments at day 0 (○), day 21 (◊), and day 42 (☐). Samples from individual donors are connected by lines.

Considering the results from the PPP, we investigated the redox homeostasis with an emphasis on glutathione metabolism (Fig 4). Results indicate that supercooled storage results in the accumulation of extracellular glutamine while preventing cystine efflux in a temperature-dependent manner with E-Sol5 (Fig 4A, B). Storage-dependent depletion of intracellular glutamine, glutamate, and reduced and oxidized glutathione (GSH and GSSG) is observed across all groups, albeit at different rates across temperatures. Still, the GSH/GSSG ratios – a marker of the cellular redox status – were better preserved in supercooled storage with E-Sol5 indicating mitigated oxidative stress. (Fig 4A, **Supplemental Fig 3**). Activation of the gamma-glutamyl-cycle and glutathione synthesis were observed across all groups as a function of storage duration, suggestive of ongoing de novo synthesis and turn-over of GSH, especially in E-Sol5. Extracellular cystine, a marker of non-apoptotic erythrocyte-specific cell death or eryptosis, accumulated predominantly at +4 °C in both additive solutions (Fig 4B).

### Supercooled storage preserves high-energy Adenylate pools

**Figure 5.**
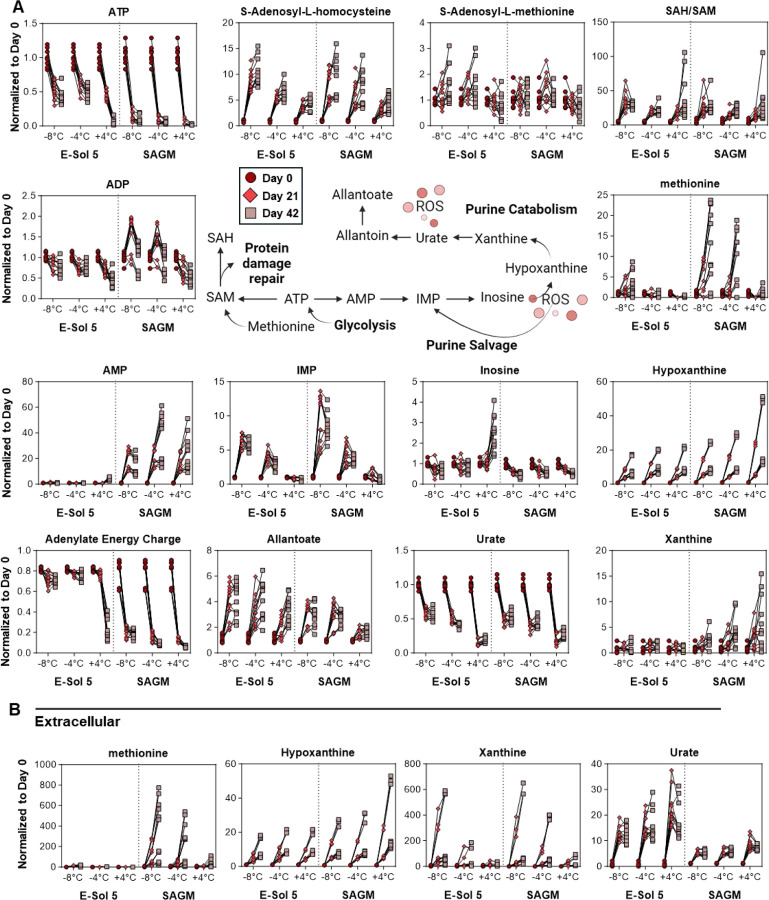
Purine Metabolism. Relative abundance measurements (normalized to respective day 0 value for each donor) for purine metabolites for both (A) intracellular and (B) extracellular compartments at day 0 (○), day 21 (◊), and day 42 (☐). Samples from individual donors are connected by lines.

Total adenylate pools and associated purine catabolism/salvage are modulated by oxidative stress^64^. Consistent with the elevated RBC metabolism, storage at all temperatures in E-Sol5 was associated with higher levels of ATP and ADP, but not AMP when compared to storage in SAGM across all time points (**Supplemental Fig 4**). The relative rates of change for ATP and ADP/AMP, respectively, were higher in RBCs in SAGM (Fig 5A). As such, Adenylate Energy Charge (AEC) was particularly well maintained in supercooled RBCs in E-Sol5, reflecting their preserved glycolytic activity (Fig 5A). Notably, supercooled storage slowed down ATP and ADP breakdown into AMP, though deamination to IMP was the highest in RBCs stored in E-Sol5 at −8 °C, followed by those in E-Sol5 at −4 °C. This is partly explained by the lowest inosine levels in these two groups, suggesting that only at +4 °C and with E-Sol5, IMP breaks down to inosine. The relatively higher ratios of IMP to inosine suggest either slower adenylate degradation or improved maintenance of purine salvage pathways at supercooled temperatures (Fig 5A). Consistent with a decrease in oxidant stress in E-Sol5, especially at supercooled temperatures, decreased ROS-mediated conversion of the antioxidant urate into allantoate was observed throughout storage (**Supplemental Fig 4**).

RBCs use the cofactor S-adenosyl-homocysteine (SAM) and Protein-L-isoaspartate O-methyltransferase (PCMT1) system, to repair proteins damaged by oxidative stress^65^. Accumulation of the product, S-adenosyl-homocysteine (SAH), was noted in the E-Sol5 group, especially at the lowest temperatures (**Supplemental Fig 4**), though the rates were comparable with SAGM (Fig 5A). These suggest that despite decreased utilization of urate and GSH as ROS scavengers, supercooled RBCs stored in E-Sol5 may leverage the PCMT1 system to repair protein damage. Alternatively, unexpected (given recent literature in AS-3^65^), methionine accumulation was observed in supernatants across all groups except E-Sol5 at +4 °C (**Supplemental Fig 4**). The highest respective elevations of methionine were noted at −8 °C in both additives at the end of storage, followed by storage at −4 °C in SAGM only. This result suggests potential proteolysis-derived elevation in the levels of this amino acid (Fig 5).

### Supercooling prevents the accumulation of free fatty acids and consumption of acyl-carnitines

**Figure 6.**
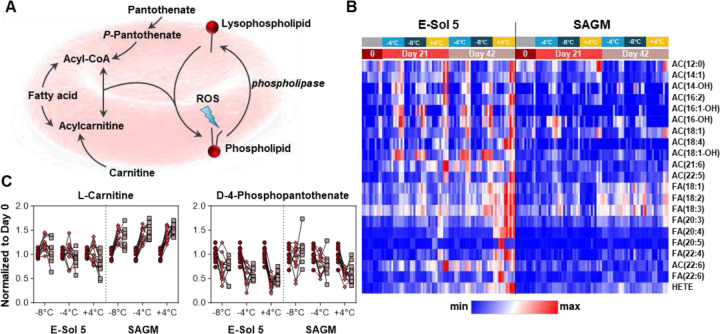
Fatty Acid and Acylcarnitine Metabolism. (A) The Lands Cycle is used by RBC for membrane repair. **(B)** Relative abundance measurements (in peak area top, arbitrary units) for cofactors carnitine and pantothenate-phosphate at day 0 (○), day 21 (◊), and day 42 (☐). (C) Heat map depicting relative abundance of acylcarnitines (AC) and fatty acids (FA) across storage timepoints and conditions. Samples from individual donors are connected by lines.

Storage-induced and oxidant stress-dependent activation of phospholipase A2 (PLA2) or PLA2-like enzymes such as peroxiredoxin 6^66,67^ has been observed during RBC storage and represents a hallmark of storage-induced lipid peroxidation (Fig 6A). In this view, the elevation of long and very-long chain poly- and highly-unsaturated fatty acids (*i.e.*, FA 18:3 to 22:6) was observed here as a function of storage time across all conditions, with the strongest effect in RBCs stored with E-Sol5 at +4 °C. Notably, these effects were attenuated or even abolished by supercooled storage, especially in E-Sol5 (Fig 6B) suggesting decreased activation of oxidant-sensitive fatty acid desaturases^68^.

Given the absence of mitochondria in mature RBCs to fuel beta-oxidation, the conjugation of free FAs and carnitine generates the acyl-carnitine pools that RBCs use to replace damaged membrane lipid acyl chains. Depletion of carnitine pools is a hallmark of RBC aging *in vivo* and *in vitro*, as these pools are essential to fuel the Lands cycle^36^. Notably, storage in E-Sol5 led to increased acyl-carnitine (AC) pools in RBCs. However, the effect of donor biology (especially sex dimorphism) on L-carnitine pools (**Supplemental Fig 5C**) was stronger than that of storage additives and temperatures. Ultimately, we measured substantial accumulation of the arachidonic acid peroxidation end product – hydroxyeicosatetraenoic acid (HETE – isomers) in RBCs stored in both E-Sol5 and to a lesser extent in SAGM, predominantly at +4 °C (Fig 6B). In E-Sol5, but not in SAGM, we observed a storage-dependent depletion of phosphopantothenate (**Supplemental Fig 5C**), a precursor to CoA (acyl-CoAs and acyl-carnitines are in equilibrium in the Lands cycle). Altogether, these results suggest increased lipid peroxidation and membrane lipid turnover in cold-stored RBCs, a phenomenon notably mitigated by supercooled storage.

## Discussion

Supercooled storage is a novel method with a premise of extending storage time of banked donor RBCs, with a goal of extension to >100 days. This premise is based on the notion that subzero temperatures in supercooled storage can substantially slow down metabolism to mitigate the metabolic storage lesion and thus extend storage time. Recently, we demonstrated this premise where we showed that only alkaline additive solutions, among which E-Sol5 stood out, provided hemolysis comparable to cold storage, while conventional solutions, especially SAGM, resulted in significant hemolysis^58^. These results necessitated a detailed understanding of the unique injuries and metabolic benefits of supercooled storage of RBCs such that we can subsequently improve it towards high-quality and long-term storage. In this regard, metabolomics approaches provide a wealth of data and pathway-level understanding of RBC metabolism. Such metabolomics approaches have already contributed to advancing our understanding of the mechanisms underlying the onset, progression, and severity of the storage lesion^45,69^, as a function of storage temperatures^12^ and the composition of storage additives and paved the way for developments in the RBC storage practice^11,45,70–72^.

Combining measurements of hemolysis and high-throughput metabolomics, we report that storage of RBCs, independent of storage temperature, in alkaline additive E-Sol5 has beneficial effects that include increased activation of glycolysis, the PPP and glutathione homeostasis, ultimately preserving high energy adenylate pools better than SAGM. Importantly, we show that supercooled storage slows down RBC metabolism manifesting as decreased glucose consumption and lactate accumulation, and better conservation of adenylate pools and 2,3-DPG levels than cold storage. These impacts were especially notable with the alkaline additive solution, E-Sol5. The 2,3-DPG conservation is particularly striking given its importance for functional and therapeutic success in the transfusion of stored RBCs, especially in critically ill patients with compromised vasculature^73^. While higher levels of 2,3-DPG throughout storage are in part driven by alkaline conditions of E-Sol5, supercooled storage of RBCs significantly enhanced this effect towards the end of storage. During storage, 2,3-DPG is consumed to generate ATP for the maintenance of other functions requisite in RBC homeostasis. One such process that demands large amounts of ATP is ion homeostasis maintained through the activity of ATP-dependent Na^+^-K^+^ pumps^74^. As the kinetics of these transporters slow with decreasing temperature^75^, it is possible that supercooled storage of RBCs decreases the demand for ATP-mediated ion homeostasis, thereby maintaining ATP levels and obviating 2,3-DPG catabolism. Ion levels including sodium, potassium, and calcium, all of which modulate RBC function and affect hemolysis, are known to change with storage^72^. Accordingly, future work will also focus on the impact of supercooled storage on ion homeostasis. Overall, supercooled storage of RBCs in E-Sol5 prevents the catabolism observed in both cold storage and supercooled storage in SAGM, throughout the whole storage duration.

We also report that supercooled storage, at both temperatures, preserves reduced to oxidized glutathione (GSH/GSSG) ratios better than standard cold storage, especially in E-Sol5, indicative of ameliorated oxidative stress. In agreement, supercooled RBCs also presented lower levels of free fatty acids and elevated total acyl-carnitine pools especially when stored in E-Sol5. These findings are indicative of increased membrane repair capacity and are consistent with a decrease in hemolytic propensity in supercooled RBCs when stored in this additive as opposed to standard SAGM. Significantly differential pathways also point to enhanced methionine consumption and SAH elevation in supercooled E-Sol5 storage. This signature suggests increased activation of protein damage repair mechanisms catalyzed by enzymes like PCMT1, which responds to oxidant stress-induced dehydration and deamidation of aspartate and asparagine residues, respectively^65^. Despite improvements in small molecule antioxidant levels, a putative upregulation in the PCMT1 system might suggest proteome-specific oxidant stress in E-Sol5 and at supercooled temperatures that is alleviated by methionine-fueled pathways. Future studies using proteomics^65^ will investigate this axis and the potential for methionine supplementation^60^ to fuel protein damage repair.

This study holds several limitations. First, multiple variables such as storage temperatures, additive solutions, and duration were tested, thus requiring us to split units into smaller volumes. Further, the use of packed RBC units from a commercial vendor and the subsequent splitting necessitated washing of the RBCs prior to storage such that the initial hemolysis of the stored samples were below 0.2% which is not standard practice in blood banking. Additionally, while we stored RBCs at a hematocrit of ~50%, standard banking practices use ~70% hematocrit. Overall, while the present study represents valuable proof of feasibility and the first-of-its-kind discovery of metabolic changes at supercooled temperatures, further studies are necessary to scale up the present investigation into adequately sized routine blood bags. Given our focus on metabolic changes as a function of time, temperatures, and additive solutions, other relevant variables such as donor biology, including age or genetic ancestry were not tested. Yet, despite the relatively small scale of the study, donor sexes were equally accounted for as relevant biological variables, with an observed minimal impact (especially on carnitine metabolism) compared to that of storage temperatures and additives in this pilot analysis.

Despite these limitations, our results suggest that supercooled RBCs stored at −4 °C in E-Sol5 represent a good compromise between a sufficiently slower metabolism that preserves both energy and antioxidant reservoirs, without inducing excess hemolysis observed at −8 °C. Importantly, as an immediate clinical insight, our studies show that contrary to the FDA guidance which mandates operation between 1–6 °C, RBCs can survive lower temperatures. This result necessitates i) potential reconsideration of allowable temperature ranges or allowing transient fluctuations below 1 °C during RBC storage; and ii) research into the viability of RBC storage around 0 °C (−2 °C to 1 °C) which might be achieved using existing blood banking infrastructure worldwide. Further, in light of our results and the metabolomics/hemolysis trends observed as part of our time course analyses, future studies are warranted to improve on the base alkaline solution used here and to address the feasibility of supercooled RBC storage for periods longer than currently allowed product shelf-lives. We aim to undertake such studies and contribute to improving both RBC banking practices and stable inventorying of modified RBC products in the future.

## Data Availability

The source files for the data will be made available to all interested parties upon request. Interested parties should reach out to the corresponding authors (Dr. Jason P. Acker, Dr. Angelo D’Alessandro, or Dr. O. Berk Usta) listed. The authors will also make these data sets available through select public repositories upon publication.
